# Expanding our understanding of (mal)adapted stress physiology in psychiatric disorders: achieving single-cell characterisation of steroids and neuropeptides

**DOI:** 10.1016/j.ynstr.2025.100739

**Published:** 2025-06-06

**Authors:** Katrina Z. Edmond, Natalie Matosin

**Affiliations:** a School of Medical Sciences, The University of Sydney, NSW, 2050, Australia; b Charles Perkins Centre, The University of Sydney, NSW, 2050, Australia; c Brain and Mind Centre, The University of Sydney, NSW, 2050, Australia; d Molecular Horizons, School of Science, Faculty of Science, Medicine and Health, University of Wollongong, Northfields Ave, Wollongong, 2522, Australia

**Keywords:** Psychological stress, Psychiatric disorders, Neurosteroid, Neuropeptide, Spatial analysis, Single-cell, Multiomic analysis

## Abstract

Steroid hormones and neurosteroids (collectively neuroactive steroids), alongside neuropeptides, are key modulators of the central nervous system. These signalling molecules integrate environmental cues into neurobiological responses by regulating gene and protein expression in a cell-type-specific manner. Specifically, neuroactive steroids and neuropeptides modulate the hypothalamic-pituitary-adrenal axis to influence excitatory/inhibitory balance in the brain and broadly impact mood, cognition, and memory. Despite their central role in brain function, these signalling systems remain historically understudied, exposing a major gap in our understanding of stress-related psychiatric disorders, and posing a valuable opportunity for therapeutic innovation. Foundational studies using histology, genetic manipulation, and bulk transcriptomic approaches, primarily in rodent models, have provided critical insights into their roles. However, these traditional methods lack the resolution to capture region- and cell-specific mechanisms, which are needed to develop precision medicine approaches. The emergence of single-cell and spatial technologies now offers unprecedented insight into the precise cellular, molecular and spatial context in which neuroactive steroid and neuropeptide signalling occurs. By moving beyond cell-type-averaged measures, these tools enable detailed mapping of transcriptional and proteomic changes across specific brain areas and cell-types, helping to identify the microenvironments in which these systems become dysregulated. This review synthesises current knowledge of neuroactive steroids and neuropeptides in stress biology and psychiatric illness and discusses how cutting-edge molecular profiling technologies are beginning to transform our ability to study, and therapeutically target, this complex and dynamic neuroendocrine network.

## Highlights

• Steroids and neuropeptides (SNs) are key stress-responsive regulators of the CNS.
• SNs are crucial for mood regulation, cognition and memory.
• Our understanding of how stress affects SNs has come mainly from bulk approaches.
• We review cutting-edge molecular profiling technologies to study and target SNs.

## 1 Introduction

Steroid hormones and neurosteroids (together neuroactive steroids), and neuropeptides are key regulators of the central nervous system (CNS), influencing cell-type specific gene and protein expression in response to common environmental factors including psychosocial stress (Stornetta et al., 1988; Melcangi et al., 1998; Lai et al., 2000; King et al., 2002; Shen et al., 2003; Ubink et al., 2003; Agís-Balboa et al., 2006; Brown and Spencer, 2013; Locci and Pinna, 2017; Russo, 2017; Lin et al., 2022; Wu et al., 2023). As regulators of the hypothalamic-pituitary-adrenal (HPA) axis, neuroactive steroids and neuropeptides function at the interface of environmental influences, including exposure to psychosocial stressors, and neurobiological processes such as alteration to the balance of inhibitory and excitatory signalling in the brain (E/I balance; Kaul et al., 2022; Lam et al., 2022; Kos et al., 2023; Uliana et al., 2024). Together, neuroactive steroids and neuropeptides play important roles in regulating individual cells to cause net effects on neurological functions and behavioural outcomes, with impacts on mood, cognition, and memory (Brown and Spencer, 2013; Wu et al., 2023). Despite this, neuroactive steroids and neuropeptides are historically understudied, presenting a highly interesting and emerging area of investigation, and a major opportunity for therapeutic innovation.

Early pharmaceutical intervention has long been recognised as one of the most effective ways to prevent long-term disability from psychiatric illness (McGorry et al., 2010). However, most currently available treatments target the same molecular pathways as first-generation drugs (Paul and Potter, 2024), predominantly focusing on monoamines and classical neurotransmitters (Braslow and Marder, 2019). While this approach has been foundational, it has led to variable treatment efficacy, with only certain patient subgroups responding to treatment (Yoshida and Takeuchi, 2021; Howes et al., 2022), while others display low treatment compliance and negative side effects (Semahegn et al., 2020; Ghosh et al., 2022). To move beyond these limitations, there is growing interest in alternative molecular systems that can offer greater specificity and improved outcomes for patients.

In this context, neuroactive steroids and neuropeptides are emerging as promising candidates (Girdler and Klatzkin, 2007; Cohen et al., 2012; Vollmer et al., 2016; Nandam et al., 2020). Preclinical studies in rodent models have shown that these molecules are central to the regulation of stress-related neurobiology, including modulation of the HPA axis and maintenance of E/I balance in the brain. Crucially, they also reveal clear sex differences in stress vulnerability and resilience, offering a biological basis for individual variation in psychiatric risk and treatment response (Ménard et al., 2016; Brivio et al., 2023; Van Doeselaar et al., 2025). Translational evidence from human studies also supports this potential, as individuals with psychiatric disorders exhibit distinct, cell-type-specific alterations in the expression of neuropeptide genes and their receptors (Zhong et al., 2022). This suggests that HPA axis dysregulation may operate through precise molecular changes that differ by cell-type and sex, offering a map of highly specific, therapeutic entry points. However, to progress further, we need to extend our understanding of this system to the next level of upstream cell-specific regulation.

In this review, we explore the current evidence for how neuroactive steroids and neuropeptides are affected by physiological stress and discuss their role in psychiatric illness. Additionally, we focus on the steroid hormone cortisol, the neurosteroid allopregnanolone, and neuropeptide Y, as case studies to emphasise the need for techniques capable of facilitating spatially resolved analysis of upstream stress regulatory networks at the cell-specific level. Overall, we advocate for the use of these technologies to further our investigation into neuroactive steroids and neuropeptides, specifically how they interact as upstream regulators of dysregulated stress signalling. Together, this has the potential to redefine how we understand changes to these molecules as a risk factor, and as potential biologically based treatment targets, for stress-related psychiatric phenotypes.

## 2 Neuroactive steroid mechanisms

Neuroactive steroids mainly exert their effects through the HPA axis, which is a highly coordinated, neuroendocrine system comprised of the hypothalamus, pituitary and adrenal glands (Crowley and Girdler, 2014). The HPA axis helps regulate several physiological processes in response to environmental stimuli, including physiological stress signalling (McEwen et al., 2015; Godoy et al., 2018; Sheng et al., 2021). When stimulated, gamma-aminobutyric acid (GABA)ergic signals from the paraventricular nucleus (PVN) of the hypothalamus elicit the secretion of corticotropin-releasing hormone (CRH) into the hypophyseal portal system (Juruena et al., 2004; Cullinan et al., 2008). CRH then stimulates the secretion of adrenocorticotropin hormone (ACTH) from the anterior pituitary gland, which initiates the release of glucocorticoid hormones, principally cortisol, from the adrenal glands (McEwen et al., 2015). Since cortisol is lipid permeable, it can easily diffuse across cellular membranes to influence cell-specific gene expression and facilitate cells to physiologically adapt to the stimulus (Souza-Talarico et al., 2011; Russo et al., 2012; Meijer et al., 2019).

Although both are steroids, neuroactive steroids differ from steroid hormones in their broad mechanism of action. Under basal conditions, steroid hormones such as cortisol, bind to mineralocorticoid receptors whose expression is particularly enriched in limbic structures such as the hippocampus (Reul and De Kloet, 1985; Ramamoorthy and Cidlowski, 2016). During periods of sustained stress signalling, these receptors become saturated, and cortisol shifts to signal through glucocorticoid receptors, which are more broadly expressed throughout the CNS (Reul and De Kloet, 1985; Reul et al., 1987). Since both mineralocorticoid and glucocorticoid receptors are intracellular hormone receptors, they both contribute to regulation of the same functions, such as memory retrieval and consolidation (Harris et al., 2013). However, studies also suggest that glucocorticoids can mediate independent, sometimes opposing, transcriptional responses (Groch et al., 2013; Koning et al., 2019). While the full mechanism driving this cell-type specificity remains unclear (Joëls, 2006; Koning et al., 2019), there is evidence that it may be governed by chromatin organisation and coregulator recruitment (Clarisse et al., 2024). In the context of stress, expression of both glucocorticoid and mineralocorticoid receptors is highly tissue- and context-specific (Joëls, 2006; Koning et al., 2019; Cruceanu et al., 2022), and their spatial localisation is therefore highly significant.

**Fig. 1:**
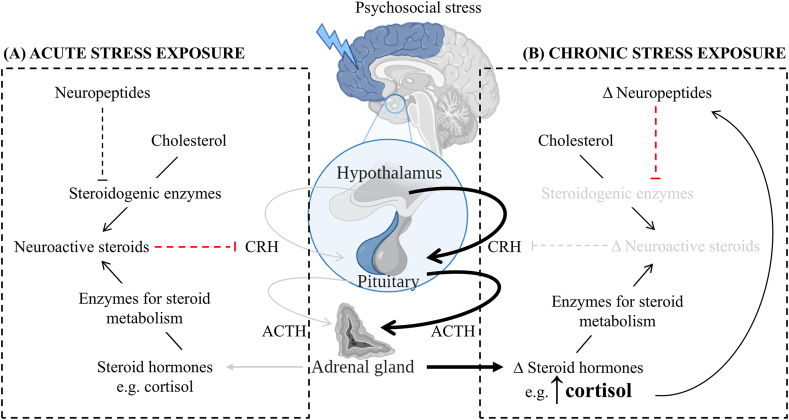
**Schematic representation of how stress activates the HPA axis, including the involvement of neuropeptides and neuroactive steroids as modulators of negative feedback.** Both panels depict the HPA axis as we currently understand it, a cascade of endocrine signalling (indicated by solid lines) that elicits the production of steroid hormones, including cortisol, from the adrenal glands. Peripheral steroid hormones can be enzymatically converted to neurosteroids, which together with cholesterol-derived neurosteroids synthesised *de novo* in the brain (which themselves are regulated by stress responsive neuropeptides) assist in HPA axis regulation via negative feedback. Panel (A) outlines how the system is thought to self-regulate following acute exposure to severe stress, that is neuroactive steroids feedback to inhibit (indicated by red dotted line) the production of CRH, returning the system to homeostatic levels (indicated by gray solid lines). Panel (B) outlines (mal)adaptation of the system following chronic exposure to stress. Specifically, continued exposure to glucocorticoid signalling alters the transcriptome of cells, decreasing the availability of steroidogenic enzymes, and therefore *de novo* neurosteroid synthesis via altered neuropeptide expression (indicated by red dotted line). As there is decreased inhibition of the HPA axis, the accumulation of glucocorticoids continues (indicated by bolded black lines), and acts to produce biological changes which are hypothesised to contribute to the aetiology of psychiatric illness. Abbreviations: ACTH – adrenocorticotropic hormone; CRH – corticotropin releasing hormone; HPA – hypothalamic-pituitary-adrenal.

Regulation of the HPA axis relies heavily on several negative feedback systems (Fig. 1A). Principally, rapid local feedback occurs via membrane-associated glucocorticoid receptors whose activation suppresses excitatory synaptic inputs to the PVN (Myers et al., 2012). There is also a well-established role of GABA, the primary inhibitory signalling molecule in the brain, in regulating the HPA axis (Cullinan et al., 2008). Potentiation of GABA signalling occurs through its binding with post-synaptic GABA type A receptors (GABA_A_R; Waraich et al., 2004; Crowley and Girdler, 2014; Schweizer-Schubert et al., 2021), N-methyl-D-aspartate (NMDA), α-amino-3-hydroxy-5-methyl-4-isoxazolepropionic acid (AMPA) or kainate receptors (Burnell et al., 2019). Interactions at the GABA_A_R are particularly interesting, as several neuroactive steroids including allopregnanolone and allotetrahydrodeoxycorticosterone (ALLO-THDOC), are known positive allosteric modulators of GABA_A_R (Morrow et al., 1987; Crowley and Girdler, 2014). Specifically, allopregnanolone and ALLO-THDOC bind to allosteric sites on GABA_A_R, increasing the affinity of GABA for the receptor active site, potentiating chloride induced membrane signalling and therefore, decreased CRH secretion (Morrow et al., 1995; Belelli and Lambert, 2005). As a result, both allopregnanolone and ALLO-THDOC exert strong analgesic and anxiolytic effects (Kavaliers and Wiebe, 1987; Bitran et al., 1995; Schüle et al., 2014). Although the GABAergic and glutamatergic systems are difficult to directly target given their extensive involvement in neurotransmission, it is feasible that they may be indirectly modulated at a fine-scale by harnessing the cell-type specificity of neuroactive steroid action.

### 2.1 Acute vs chronic exposure to physiological stress: how steroids can illicit cell-type specific effects

Depending on the context of exposure to stress stimuli (e.g., whether acute or chronic), (mal)adaptation of steroid signalling can have differential cellular effects (Fig. 1). Rodent models of acute exposure to stress (e.g., swimming, foot shock or carbon dioxide exposure) show increased synthesis of neuroactive steroids and associated E/I balance disruption (Barbaccia et al., 1996, 1997; Reddy, 2010; Reddy et al., 2021). Contrarily, chronic stress exposure can decrease the expression of some neuroactive steroids to the point that they lose capacity to act as neuromodulators (for extensive review see Reddy et al., 2021, Fig. 1B). A dynamic but tightly regulated E/I balance is an essential component of neurotransmission, especially in facilitating connectivity between functionally distinct brain regions, cell populations and microenvironments (Page and Coutellier, 2019; Kaul et al., 2022). Alterations to E/I balance have been studied in the context of stress (Kos et al., 2023), and several psychiatric disorders including schizophrenia (Ferguson and Gao, 2018; Selten et al., 2018; Kaul et al., 2022), mood disorders (major depression, bipolar disorder; Duman et al., 2016; Duman et al., 2019; Kaul et al., 2022), and autism spectrum disorders (Ferguson and Gao, 2018; Selten et al., 2018), however the extent and trajectory of these imbalances remains unclear, and at times, contradictory (Page and Coutellier, 2019). As such, although decades of research has shown a strong relationship of cyclic causality between HPA axis regulation and the expression of neuroactive steroids, how this system self-regulates in response to complex environmental, genetic and biological factors is yet to be fully understood (Crowley and Girdler, 2014; Cai et al., 2018; Schweizer-Schubert et al., 2021; Pisu et al., 2022; Hantsoo et al., 2023). Thus, understanding E/I balance (dys)regulation following chronic stress, particularly in the context of sex differences and phenotypically distinct disorder presentation, presents an exciting research avenue to pursue with the application of novel analysis platforms.

### 2.2 Could neuroactive steroids provide novel targets for psychotropic treatment? Insights from the steroid hormone cortisol and the neurosteroid allopregnanolone

An increasingly compelling body of evidence shows that neuroactive steroids are fundamental to several physiological processes in the brain, including development, cognition, behaviour, neuroplasticity, neuroinflammation and stress regulation (Taves et al., 2011). To date, the expression of neuroactive steroids has been studied in response to a wide range of environmental stimuli including cyclic events such as sleep (Driver et al., 1996; Carta et al., 2018), menstruation in females (Driver et al., 1996; Carta et al., 2012) and in response to social cues from the behaviour of other animals (e.g., acoustic communication regulated testosterone synthesis in female songbirds; de Bournonville et al., 2020). It is also well established that the production and action of neuroactive steroids are strongly influenced by exposure to psychological adversity (Locci and Pinna, 2017; Cruz et al., 2019), particularly during childhood and adolescence when the brain is at its most vulnerable (Brown and Spencer, 2013). Therefore, as the (mal)adaptation of the neuroendocrine system can have lasting effects on both behaviour and brain development (Bennett et al., 2016), it presents as a key area to interrogate for the novel treatment of stress-related psychiatric illness.

#### 2.2.1 The steroid hormone cortisol

Cortisol (corticosterone in rodents) is the most well-studied steroid hormone in the context of psychiatric illness. The binding of cortisol to glucocorticoid receptors initiates a complex cascade of intracellular signalling and altered gene expression (Yurtsever et al., 2019). It is hypothesised that various psychiatric conditions including major depression, bipolar disorder, generalised anxiety disorder and post-traumatic stress disorder (PTSD), along with their comorbidities, share common pathogenic mechanisms related to stress dysregulation (Godoy et al., 2018). One of the most consistent clinical findings in subjects with major depression (Juruena et al., 2004), bipolar disorder (Lee et al., 2018) and anxiety disorders (Weinberger, 2001; Godoy et al., 2018) is peripherally measured hypercortisolemia, that is a prolonged elevation in cytosolic cortisol levels. In certain disorder phenotypes, for example major depression, the association between cortisol dysregulation and stress exposure is complex, although it appears dependent on clinical variables such as disorder severity, duration of illness and type of stress (Nandam et al., 2020). However, as noted by Zorn and colleagues (2017), sex differences may confound results. Females with major depression or anxiety show a blunting of cortisol levels following exposure to psychosocial stress, whereas males often exhibit increased cortisol levels (Zorn et al., 2017).

There is also extensive evidence that several neuronal cell-types are particularly vulnerable to the effects of sustained stress signalling. Data from both humans and mice who have been exposed to severe adversity show decreased dendritic density of cortical pyramidal neurons (Keifer et al., 2015; Young et al., 2015; Kaul et al., 2020). These changes are hypothesised to decrease the availability of excitatory synaptic connections (McKlveen et al., 2016), and result in decreased connectivity between regulatory brain regions such as the thalamus and cortex (Petreanu et al., 2009; Murrough et al., 2016). Further, recently published data shows evidence of amplified inhibitory neuron cell fate in a human neurodevelopmental organoid model following chronic dexamethasone exposure (Dony et al., 2025). A growing body of literature suggests that non-neuronal cell-types are also affected. For example, astrocytes show decreased expression of *Slcla2/3* (encodes excitatory amino acid transporters 2 and 1 (EAAT2/1), respectively) following chronic ingestion of a cortisol analogue in rodents, or exposure to early life adversity in humans (Carter et al., 2013; Kaul et al., 2024). These data supports that stress-induced changes to E/I signalling are a contributing component of psychiatric illness pathology and demonstrates the need to mechanistically understand these sex-associated processes at a region- and cell-specific resolution.

#### 2.2.2 The neurosteroid allopregnanolone

**Fig. 2:**
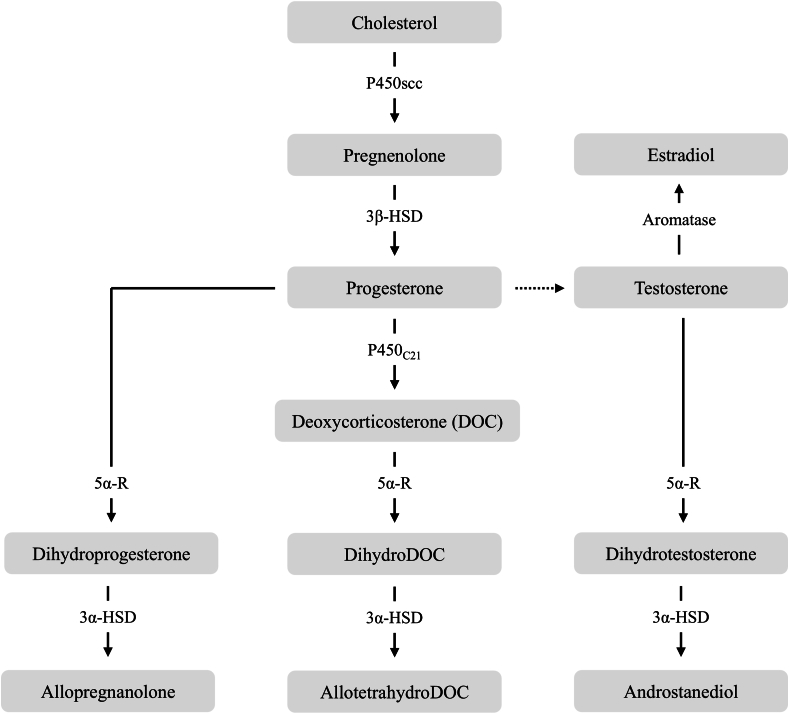
**Simplified schematic representation of neurosteroidogenesis in the vertebrate brain.** The steroidogenic enzymes cytochrome P450 side-chain cleavage (P450scc) and 3β-hydroxysteroid dehydrogenase/Δ^5^–Δ^4^ isomerase (3β-HSD) convert cholesterol into progesterone, which along with testosterone and deoxycorticosterone, act as precursors to produce terminal neurosteroids via enzymatic conversion by 5α reductase (5α-R) and 3α hydroxysteroid dehydrogenase (3α-HSD; aromatase for estradiol). Solid lines indicate direct biochemical reactions, dotted lines indicate one or more steps have been excluded for brevity.

Neurosteroids typically exert their effects on neuronal membrane receptors and ion channels, leading to a much more rapid modulation of neuronal excitability compared to steroid hormones (Burnell et al., 2019; Lloyd-Evans and Waller-Evans, 2020). This distinct mechanism has made neuroactive steroids promising targets in the development of novel, rapid-acting antidepressants (Maguire and Mennerick, 2024). The neurosteroid allopregnanolone, a derivative of progesterone (Fig. 2), is a known anxiolytic, anaesthetic agent and anticonvulsant (Schüle et al., 2014). As a potent positive allosteric modulator of GABA_A_R (Chen et al., 2021), it helps modulate the HPA axis via negative feedback (Reddy, 2006), as well as enhance neurogenesis (Wang et al., 2005) and neuroprotection (Schüle et al., 2014). Rodent models indicate that levels of both brain and peripheral plasma allopregnanolone fluctuate depending on duration of exposure to adverse stimuli, with levels rising following acute exposure and dropping following chronic exposure (Girdler and Klatzkin, 2007). In humans, reduced allopregnanolone expression has been reported in the peripheral blood and cerebrospinal fluid of individuals with stress-related psychiatric phenotypes, specifically major depression (Schüle et al., 2014), PTSD (Rasmusson et al., 2006) and the negative symptoms of schizophrenia (Marx et al., 2009). Importantly, sex differences have also been identified, with females exhibiting a distinct blunting of allopregnanolone following acute stress (Klatzkin et al., 2006; Rasmusson et al., 2006; Pisu et al., 2022).

In a preclinical male rodent model, Uzunov et al. showed that treatment with fluoxetine, a selective serotonin reuptake inhibitor (SSRI), significantly increased allopregnanolone levels in the frontal cortex in a dose- and time-dependent manner (Uzunov et al., 1996). The same authors later showed clinically that fluoxetine/fluvoxamine increased levels of allopregnanolone in the cerebrospinal fluid of human subjects with major unipolar depression, whose baseline levels were ∼60 % lower than matched controls (Uzunov et al., 1998). Notably, no changes were observed in other neuroactive progesterone derivatives or with other classes of antidepressants, such as tricyclic antidepressants (Uzunov et al., 1996, 1998; Schüle et al., 2014).

Some steroid-based therapeutic formulations, including brexanolone (a β-cyclodextrin-based formulation of allopregnanolone) have progressed to clinical trials for postpartum depression. Administered intravenously, the lipophilic nature of brexanolone allows it to readily diffuse across the blood-brain barrier where it can either interact with GABA_A_R, or reversibly convert into 5α-dihydroprogesterone and interact with progesterone receptors (Fig. 2; Rupprecht et al., 2010). Brexanolone has shown both rapid-acting and long-lasting effects on reducing depressive symptoms up to 30-days post-injection, although the mechanism for the latter remains unclear (Reddy et al., 2023). Despite this success, advancement of other steroid-based therapeutics for the treatment of stress-related psychiatric phenotypes are lacking.

Together, these data indicate that sex-specific steroid-mediated HPA axis dysregulation may contribute not only to the severity, but the phenotypic presentation of psychiatric illness in humans. As we now have evidence from both the epigenetic (Rahman and McGowan, 2022) and transcriptomic levels (Lopez et al., 2021; Cruceanu et al., 2022; Kos et al., 2023; Dony et al., 2025) that the effects of stress signalling are cell-type specific, our understanding needs to extend to the next level of regulation. Thus, to drive precision medicine approaches, a deeper characterisation of neurosteroid biosynthesis and how it is regulated upstream, is required.

## 3 The synthesis of neuroactive steroids is a complex cell-type specific process, spanning both the central nervous system and periphery

The way in which neuroactive steroids are expressed in time and space is a significant knowledge gap to close if they are to be pursued as a novel treatment option for stress-related psychiatric illness. While steroid hormones are primarily produced outside the CNS in the adrenal glands and gonads (Brown and Spencer, 2013; Amenyogbe et al., 2020), their lipophilic nature allows them to easily cross the blood-brain barrier and accumulate in the brain (Reddy, 2010). A portion of neuroactive steroids however, are synthesised as metabolites of their steroid hormone precursors while still in peripheral tissues (Reddy, 2010). Therefore, although our understanding of neuroactive steroid synthesis is well developed (for extensive review see Do Rego et al., 2009), the intricacy of this process – particularly the influence of sex differences and subjective contextual stimuli – poses a significant challenge for the study of these molecules. This complexity is particularly evident when studying clinical samples from individuals with psychiatric illness which are highly variable with many confounding factors.

In vertebrates, neurosteroidogenesis is tightly regulated by the activity of several steroidogenic enzymes, whose expressions vary across brain regions (Do Rego et al., 2009; Reddy, 2010; Taves et al., 2011) and cell-types (Melcangi et al., 1998; King et al., 2002; Agís-Balboa et al., 2006; Locci and Pinna, 2017; Lin et al., 2022). In the brain, neurosteroids are synthesised *de novo* from circulating cholesterol in principal neurons and glial cells as part of a complex biochemical pathway that involves several, often promiscuous, steroidogenic enzymes (summarised in Table 1; Miller and Flück, 2014). For example, the enzymes 5α reductase I (5α-R1) and 3α hydroxysteroid dehydrogenase (3α-HSD) are involved in the synthesis of three terminal neurosteroids, allopregnanolone, ALLO-THDOC and androstanediol (Fig. 2; Reddy, 2010). Both 5α-R1 and 3α-HSD also show cell-type specific patterns of expression. Specifically, 5α-R1 is expressed in astrocytic cells of the rat cerebral cortex and hypothalamus (Pelletier et al., 1994), while both enzymes colocalise in cortical and hippocampal glutamatergic principal neurons of the mouse brain (Agís-Balboa et al., 2006).

**Table 1:** Summary of key steroidogenic enzymes involved in the synthesis of neuroactive steroids.

Enzyme	Abbr.	Gene	Role	Evidence of expression in the human cerebral cortex
Steroidogenic acute regulatory protein	StAR	*STAR*	Regulates cholesterol transport (Clark et al., 1994)	High mRNA (Inoue et al., 2002), protein (King et al., 2002)
Cytochrome P450 side-chain cleavage	P450scc	*CYP11A1*	Cleaves side chain to convert cholesterol to pregnenolone (Lieberman et al., 1984)	Moderate/high mRNA (Watzka et al., 1999; Inoue et al., 2002), protein (King et al., 2002)
3β-hydroxysteroid dehydrogenase/Δ^5^–Δ^4^ isomerase	3β-HSD	*HSD3B2*	Catalyses conversion of Δ5-3β-hydroxysteroids into Δ4-3-ketosteroids (Do Rego et al., 2009)	Moderate mRNA (Inoue et al., 2002)
Cytochrome P450 17α-hydroxylase/C17,20-lyase	P450_C17_	*CYP17A1*	Catalyses hydroxylation of C_21_ steroids and C-17,20 bond cleavage between 17α-hydroxylated steroids to give C19 ketosteroids (Zwain and Yen, 1999)	Low/nill mRNA (Stoffel-Wagner, 2003)
17β-hydroxysteroid dehydrogenase	17β-HSD	*HSD17B3*	Catalyses interconversion of 17-ketosteroids and 17β-hydroxysteroids (Labrie et al., 2000)	Moderate mRNA (Stoffel-Wagner et al., 1999), enzymatic activity (Steckelbroeck et al., 1999)
5α reductase I	5α-R1	*SRD5A1*	Facilitates NADPH catalysed reduction of C4-C5 double bond (Lephart, 1993)	High mRNA ( Stoffel-Wagner et al., 1998b)
3α hydroxysteroid dehydrogenase	3α-HSD	*AKR1C2*	Reversibly interconverts 5^α^ reduced steroids into tetrahydroxysteroids (Do Rego et al., 2009)	High type II mRNA, low type III mRNA (Griffin and Mellon, 1999)
Cytochrome P450_7α_	P450_7α_	*CYP7B1*	Catalyses conversion of steroid precursors into hydroxylated metabolites (Myant and Mitropoulos, 1977)	Low mRNA (Yau et al., 2003)
Cytochrome P450 aromatase	P450arom	*CYP19*	Catalyses the terminal conversion of C19 androgens into C18 estrogens (Stoffel-Wagner et al., 1998a)	Moderate mRNA (Sasano et al., 1998; Stoffel-Wagner et al., 1998a) Protein (Yague et al., 2006)
Hydroxysteroid sulfotransferase	HST	*SULT2A1*	Regulates the synthesis of sulphated steroids (Do Rego et al., 2009)	–

Several other steroidogenic enzymes show cell-type specific expression patterns in the brain. Specifically, extensive evidence from enzymatic activity assays, transcript and/or protein expression in the human cerebral cortex using bulk analysis of surgically resected (Stoffel-Wagner et al., 1998a, Stoffel-Wagner et al., 1998b; Steckelbroeck et al., 1999; Stoffel-Wagner et al., 1999; Watzka et al., 1999; Inoue et al., 2002; Stoffel-Wagner, 2003) and postmortem tissues (Sasano et al., 1998; Griffin and Mellon, 1999; King et al., 2002; Yau et al., 2003; Yague et al., 2006) show that the majority of steroidogenic enzymes demonstrate moderate to high expression in the human cerebral cortex, apart from Cytochrome P450 17α-hydroxylase/C17,20-lyase (Stoffel-Wagner, 2003) and Cytochrome P4507α (Yau et al., 2003), which show low to negligible expression (Table 1). Detailed characterisation of sex-specific changes to the expression of steroidogenic enzymes is still required.

Given the existence of several physiologically distinct pathways that sustain the synthesis of neuroactive steroids in the human brain, it could be hypothesised that idiosyncratic (mal)adaptations across these pathways may contribute to the same overarching phenotype of psychiatric illness. For example, Griffin and Mellon show that multiple forms of 3α-HSD are present within the human brain, each with distinct enzymatic activity profiles (Griffin and Mellon, 1999). Their data suggests that two of these isoforms are sensitive to SSRI action and demonstrate expression profiles which are specific across brain regions. They hypothesise that SSRIs may influence neurosteroid production differently across the brain, providing a potential mechanism for modulating patient-specific behaviours (Griffin and Mellon, 1999).

## 4 What is regulating the regulators? Neuropeptides as proposed modulators of *de novo* neurosteroid synthesis

Neuropeptides have been proposed as modulators of *de novo* neurosteroid synthesis in the brain (Fig. 1; Ubuka and Tsutsui, 2022), and alteration in neuropeptide expression (mal)adaptations are hypothesised to be key factors in the pathoetiology of stress-related psychiatric illness (Suchak et al., 2022). Evidence shows that neuropeptides play a critical role in modulating the physiological stress response, specifically through regulating the expression and activity of several steroidogenic enzymes (Ubuka and Tsutsui, 2022; Wu et al., 2023). As such, chronic exposure to stress can lead to (mal)adaptative changes in this system, whereby the expression and biological actions of neuropeptides shift in a context dependent manner (Fig. 1B; Kormos and Gaszner, 2013). For example, it has been hypothesised that glucocorticoid-induced transcriptomic changes such as altered neuropeptide expression increase the inhibitory regulation of steroidogenic enzymes, leading to altered rates of *de novo* neurosteroid synthesis (Do Rego et al., 2009; Ubuka and Tsutsui, 2022; Meijer et al., 2023). Consequently, altered neurosteroid synthesis contributes to an overall decrease in HPA axis inhibition, leading to the accumulation of glucocorticoids, such as cortisol (Crowley and Girdler, 2014). If the HPA axis remains disinhibited for long periods, it is hypothesised to permanently alter the structural and functional biology of cells, therefore increasing an individual's risk for developing a psychiatric phenotype (Fig. 1B; McEwen et al., 2016; Dziurkowska and Wesolowski, 2021). Thus, to fully disentangle this association, the next critical step is to deeply characterise the neuropeptidergic regulatory mechanisms that govern neurosteroid biosynthesis in a sex- and cell-type specific manner.

### 4.1 Neuropeptide synthesis is cell-type specific in the vertebrate brain

**Fig. 3:**
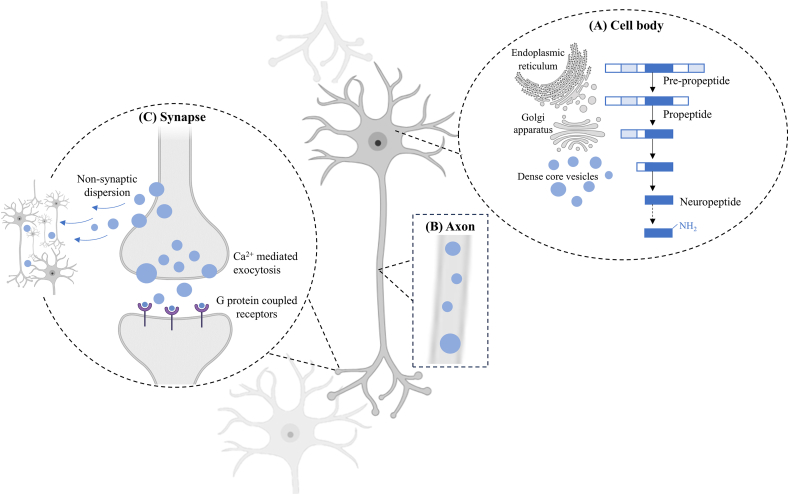
**Schematic representation of neuropeptide processing and release within neuronal cells.** (A) Within the cell body, pre-propeptides are synthesised in the endoplasmic reticulum and following cleavage of the signal peptide sequence, become propeptides. As the propeptides make their way through the Golgi apparatus, they undergo proteolytic cleavage which continues within the dense core vesicles. Some neuropeptides, but not all, undergo additional modifications including glycosylation and/or C-terminal amidation. (B) The dense core vesicles, now containing biologically active neuropeptides, traverse the axon towards the synapse. (C) Once at the synapse, the dense core vesicles can be released via either Ca^2+^ mediated exocytosis to act on local G protein-coupled receptors or undergo non-synaptic dispersion into the extracellular fluid to travel and act on distant targets.

In the human brain, neuropeptides represent the most diverse class of neurotransmitters, participating in autocrine, paracrine (van den Pol, 2012), and endocrine signalling (Russo, 2017), with >100 members currently identified (Zhong et al., 2022). Neuropeptides are synthesised within the CNS and are almost always co-localised with one or more small molecule neurotransmitters (Fig. 3; van den Pol, 2012; Hökfelt et al., 2018). The synthesis of neuropeptides in neurons is well-documented and begins in the cell soma with the production of pre-propeptides in the endoplasmic reticulum (Russo, 2017). During this initial stage, cleavage of the signal peptide sequence results in propeptides (Hook et al., 2008). The propeptides then undergo sequential proteolytic cleavage as they pass through the Golgi apparatus and are packaged into dense core vesicles (Fig. 3A; Hook et al., 2008).

To become biologically active, some, but not all, neuropeptides undergo additional modifications including glycosylation and/or C-terminal amidation (Fig. 3A; Eipper et al., 1992; Liu et al., 2019). The dense core vesicles, now containing biologically active neuropeptides, are then trafficked along the neuronal axon, towards the synapse (Fig. 3B; Russo, 2017). Upon reaching the synapse, the vesicles can be released via calcium (Ca^2+^) mediated exocytosis to activate local G protein-coupled receptors (GPCRs) or undergo non-synaptic dispersion into the extracellular fluid, allowing them to act on distant targets (Fig. 3C; Hook et al., 2008; Russo, 2017). Activation of GPCRs induce a conformational change in the receptor complex, triggering downstream effects within the cell (Hazell et al., 2012; van den Pol, 2012). One such pathway involves the modulation of ion channels that regulate neuronal activity and action potential probability within neuronal networks (van den Pol, 2012). Beyond neuronal modulation, neuropeptides have been shown to influence the activity of glial cells including microglia (Carniglia et al., 2017), astrocytes (Asrican et al., 2020) and oligodendrocytes (Osso et al., 2021).

Compared with neurons, considerably less is known about the synthesis and release of neuropeptides from glia (Ubink et al., 2003), although neuropeptides are also reportedly expressed in microglia (Lai et al., 2000), astrocytes (Stornetta et al., 1988; Shinoda et al., 1989) and oligodendrocytes (Shen et al., 2003; Ubink et al., 2003; Ramamoorthy and Whim, 2008). Transcript presence or peptide immunoreactivity has been assessed in *in vivo* rodent models (Shen et al., 2003), cell culture studies using both rodent and human derived cell lines (Shinoda et al., 1989; Lai et al., 2000) and resected tissue culture experiments in rodents (Just et al., 1998), which collectively provides foundational understanding of how neuropeptides are expressed in the brain (Ubink et al., 2003). While it is understood that pathological processes such as the development of psychiatric illness can influence glial expression of neuropeptides (Polazzi and Contestabile, 2002), there are also many examples where expression profiles of neuropeptides in cultured glial cells do not align with those observed in resting glia from the adult human brain (Ubink et al., 2003). Nevertheless, the evidence for glial expression of neuropeptides, and the growing recognition of the critical role that reciprocal neuron-glia interactions have on information processing and signal transduction within the brain (Kato et al., 2013), together underscores the far-reaching implications of neuropeptide (mal)adaptation in maintaining neurotypical brain function.

It has also been proposed that neuropeptides make an important contribution in stabilising circuit connectivity throughout the brain (Guillaumin and Burdakov, 2021). A key feature of neuropeptide synthesis is that the sequential cleavage of precursor propeptides can generate a diverse array of unique neuropeptides from a single gene (Russo, 2017), each of which may be pleiotropic in function (Hoffmann and Williams, 2020). Thus, while evidence suggests that a wide range of neuropeptides mediate the cellular stress response, the exact mechanism and subcellular localisation of this mediation is unclear despite recent technological advancements that allow us to determine these intricacies (Carrasco and Van de Kar, 2003; Madaan and Wilson, 2009; Alldredge, 2010; Schank et al., 2012). One informative case study which exemplifies these complexities in the context of stress regulation and psychiatric illness is Neuropeptide Y.

### 4.2 Neuropeptide Y plays a major role in the physiology of cellular stress and resilience

Neuropeptide tyrosine (neuropeptide Y; NPY) is a 36 amino acid neuropeptide that is highly conserved between species and widely expressed throughout the CNS (Vollmer et al., 2016). It is reported to be implicated in a broad range of physiological processes, including energy homeostasis (White, 1993), circadian rhythms (Stanley and Thomas, 1993), feeding regulation (Stanley and Thomas, 1993; Krysiak et al., 1999), neuronal excitability (Colmers and Bleakman, 1994) and cognition (Redrobe et al., 1999). Further, due to its implication in stress physiology (Krysiak et al., 1999; Kormos and Gaszner, 2013), NPY (mal)adaptation has been investigated in the context of stress and psychiatric illness, particularly disorders characterised by depressive and anxiogenic behaviours.

Previous research has examined gene expression of *NPY* and the NPY protein, and to a lesser extent its related receptors (NPY receptors 1–6; NPY[1–6]R) in various cortical regions (Caberlotto, 2001; Mellios et al., 2009; Fung et al., 2010; McGuire et al., 2011; Vollmer et al., 2016; Sharma et al., 2022; Brancato et al., 2023). In the neurotypical human brain, transcript studies show that *NPY* and *NPY1R* mRNA expression negatively correlates with age, with the most significant decline occurring during the first few years of postnatal life (Caberlotto, 2001; Fung et al., 2010). Moreover, research in rodent models has demonstrated an increase in NPY protein levels in the prefrontal cortex following unpredictable stress paradigms (such as restraint, hypoxia, forced swimming, cold exposure, or temporary crowding; McGuire et al., 2011), as well as binge-like alcohol administration during adolescence (Brancato et al., 2023). While these findings suggest that NPY signalling (mal)adaption plays a role in altered cellular physiology after stress exposure, the precise mechanisms and specific brain circuits where this occurs remain to be fully understood.

### 4.3 Expression of Neuropeptide Y and its receptors show psychiatric phenotype specificity in the cerebral cortex

Bulk analysis of *NPY* mRNA expression across several psychiatric phenotypes reveals decreased *NPY* gene expression within key regions of the limbic system. Notably, decreased *NPY* gene expression has been observed in the prefrontal cortex of humans with depression who died by suicide (Sharma et al., 2022) and in individuals with schizophrenia (Mellios et al., 2009; Fung et al., 2010). In contrast, transcriptomic analysis of *NPY* receptor mRNA shows no significant difference in *NPY1R* or *NPY2R* expression between schizophrenia, bipolar disorder or major depression in the human prefrontal cortex (Caberlotto, 2001). However, increased expression of both *NPY1R* and *NPY2R* mRNA has been reported in the prefrontal cortex of human depressed-suicide subjects (Sharma et al., 2022). These results align with earlier findings showing increased *NPY2R* mRNA in cortical layer IV of human subjects with suicide as a cause of death (Caberlotto, 2001). These findings are consistent with the current hypothesis that while the presence of psychiatric illness increases risk of suicidal behaviour (Gonda et al., 2023), suicidality itself may represent a distinct neurobiological process that should be studied separately from mood, psychotic and personality disorders (Pandey, 2013).

Disorder-specific protein analysis of NPY expression follows a similar trend, with decreased levels of NPY protein in prefrontal cortex cells of human depressed-suicide subjects compared to controls (Sharma et al., 2022). These findings have been supported by a rodent model of male PTSD, where reduced NPY levels were associated with increased behavioural disruption, a phenotype that could be restored following administration of an NPY1R agonist (Cohen et al., 2012). In contrast, Vollmer et al. found that infusion of NPY into the infralimbic cortex did not affect the neuroendocrine stress response, depression-like behaviour or working memory of male rats (Vollmer et al., 2016).

These contrasting findings suggest that although decreased gene expression of *NPY* and its protein is consistently observed across various psychiatric phenotypes, the region-specific alterations in NPY may contribute differently to the phenotypic expression of each disorder. These findings highlight the need for higher-resolution analysis of neuropeptides that can be applied across independent brain regions and cell-types to better understand the mechanistic role of neuropeptides in complex brain circuitry. Further, such analyses should explore how neuropeptide expression and localisation is changed at the cell-type level following both acute and chronic stress exposures, as these changes could provide critical insights into the phenotypic presentation of psychiatric illness.

### 4.4 Shifting towards single-cell analysis of neuropeptides

Recent advances in single-cell transcriptomics have opened new avenues for understanding neuropeptide localisation and function at a much higher resolution than traditional histological or bulk-tissue methods. Historically, studies have been limited to region-specific expression patterns, which often obscure finer details about the cellular and circuit-level roles of neuropeptides. The increased accessibility of single-cell RNA sequencing technologies has allowed for a deeper exploration of how neuropeptides are distributed across different cell-types, shedding light on their complex roles in brain function.

The most extensive characterisation to date in the human brain has utilised publicly available single-cell transcriptomic datasets to map peptidergic systems across multiple brain regions, including 17 subregions of the human prefrontal cortex as well as three reference cortices: frontal, parietal, and temporal (Zhong et al., 2022). When analysing the gene expression of *NPY* mRNA, Zhong and colleagues found that it was highly expressed in GABAergic interneurons, particularly within the anterior cingulate cortex, orbitofrontal cortex and frontal gyrus. This indicates that NPY plays a significant role in regulating inhibitory interneuron function across these regions, which are crucial for higher cognitive processes, emotional regulation and behaviour (Stevens et al., 2011; Jonker et al., 2014).

Further investigation into NPY receptors revealed notable differences in expression patterns: *NPY1R* mRNA was expressed at 10-fold higher levels in both glutamatergic and GABAergic neurons compared to *NPY2R*, which was exclusively expressed in glutamatergic neurons (Zhong et al., 2022). These findings align with earlier evidence which localised NPY1R expression to pyramidal projection neurons and GAD-67-positive interneurons in the infralimbic cortex, a key region involved in stress and emotional regulation (Vollmer et al., 2016). Similar single-cell transcriptomic methods have also been applied in rodent models (Smith et al., 2019), allowing for cross-species comparisons and deeper insights into how neuropeptide signalling might contribute to stress dysregulation across different neural circuits. These advancements not only enhance our understanding of neuropeptide distribution in the brain, but also underscore the importance of considering the dynamic interactions between cell-types and neuropeptide signalling in neuropsychiatric disorders. Understanding neuropeptide localisation at the single-cell level is a crucial step toward unravelling the complex mechanisms behind stress regulation and its maladaptive alterations in severe psychiatric disorders.

Although informative, previous studies conducted using bulk tissue homogenates or focusing only on behavioural paradigms lack the cellular resolution needed to uncover the mechanistic details underlying the biological actions of steroids and neuropeptides. Zhong and colleagues developed a methodological approach that begins to bridge this gap by offering a cell-type-specific analysis of neuropeptide expression in regions of the human prefrontal cortex. The specific cell-types and anatomical context in which sex-specific maladaptation of steroids and neuropeptides occurs is an important future step, not only in the neurotypical brain but also in the context of stress regulation and psychopathology.

## 5 Disentangling the mechanistic interactions of neuroactive steroids and neuropeptides is confounded by several of their innate structural and chemical properties

There are several technical considerations regarding the measurement of neuroactive steroids and neuropeptides which have confined their analysis to predominantly bulk and ‘destructive’ methods (i.e. methods which result in the loss of anatomical information). Many of the techniques previously used are no longer sufficient for fully recapitulating the neurobiological mechanisms underlying stress regulation in the human brain, particularly when the interactions of interest span different molecular classes.

Past studies focused on the analysis of neuroactive steroids have relied on bulk and destructive transcript analysis, receptor and steroidogenic enzyme assays, or chromatographic analysis of peripheral samples such as hair, serum, urine or saliva (Taves et al., 2011). A key limiting factor for the measurement of steroids in the brain is their low *in vivo* concentrations (Gomez-Sanchez et al., 2005), the high spatial heterogeneity of their regional expression (Charlier et al., 2010), their dynamic production and metabolism rates (Balthazart and Ball, 2006), and the synthesis of unique steroids not found in the periphery (Toran-Allerand et al., 2005). Additionally, as cholesterol derivatives, steroids are highly lipophilic, enabling them to diffuse freely across cellular membranes. This characteristic makes assay-based methods susceptible to interference from the high lipid content of brain tissue (Taves et al., 2011).

For neuropeptides, biological activation depends on several proteolytic processing and post-translational modification steps (Fig. 3A; Hook et al., 2008; Russo, 2017). This process is dynamic and challenging to study *in vivo*, as for many neuropeptides, each stage of modification has the potential to produce a unique peptide (Delaney et al., 2018). Further, the specific intracellular locations where these modifications occur can vary even among neuropeptides of the same class (Delaney et al., 2018). This complexity exemplifies one of the intrinsic characteristics that make neuropeptides more challenging to study compared to other biomolecules, such as neurotransmitters and metabolites (Delaney et al., 2018). Moreover, evidence suggests that neuropeptides can exist in multiple active isoforms, sometimes with the same chemical structure, but perform distinct functions depending on their expression relative to neighbouring cell-types or local receptor availability (Morimoto et al., 2008). Low *in vivo* concentrations (Romanova and Sweedler, 2015) and their susceptibility for rapid degradation (Schrader et al., 2014) further complicate their study.

While the structure of many neuropeptides are well conserved across mammalian species, their distribution patterns (Dumont et al., 1998) and functional properties, particularly as it relates to behavioural phenotypes, show discrepancies between studies (Althammer et al., 2018). These differences, coupled with intrinsic cytoarchitectural variations between rodent and primate brains (e.g., the lack of higher cortical structures in rodents), influence the translatability of findings and complicate efforts to synthesise mechanistic findings. As a result, data often become convoluted with conflicting evidence, shaped by a host of dynamic variables such as region specificity, disorder phenotype, sex and age. These factors can complicate the interpretation of findings and underscore the need for more nuanced, cross-species studies to resolve the complex role of neuropeptides in brain function and psychiatric illness.

Finally, although the brain is where psychiatric symptoms manifest and psychotropic medications likely exert their effects, peripheral samples offer the advantage of being measurable in living subjects. Our next step is to probe how peripheral changes *in vivo* reflect observed changes in the brain, as this may uncover biomarkers that can be used for diagnosis, intervention or personalised medicine development (Zannas et al., 2016). However, due to the multi-layer complexity of how neurosteroids and neuropeptides are synthesised, this level of understanding cannot be achieved by focusing only on discrete elements of the broader regulatory system.

## 6 Moving forward: application of high resolution, spatially resolved analysis platforms

### 6.1 Understanding gene expression landscapes

The marked spatial heterogeneity of the brain means that homeostatic pathways (and the ways in which prolonged stress or psychiatric illness can remodel them) are altered at a fine anatomical resolution. Spatial omics approaches now make it possible to pinpoint where neuropeptide precursors and steroidogenic enzyme transcripts are expressed, revealing sex and cell-(sub)type specific patterns that were previously obscured. Although many of these methods remain costly, they have become broadly accessible and are increasingly used to illuminate the regional and often time-sensitive biology of neuroactive signalling molecules (Curry et al., 2024; Ye et al., 2024). While this review is focused on the application of spatial omics to address a specific biological question, we have also recently reviewed the technical comparison of currently available commercial spatial omics platforms (Curry et al., 2024).

Stress-related psychiatric disorders appear to reshape gene expression landscapes in a highly regional and cell subtype-specific manner. For instance, in our recent study we spatially mapped mRNA profiles across the cell layers of the human orbitofrontal cortex, and then linked each spot to its corresponding single-nucleus transcriptomic signature. This showed a selective down regulation of glutamate-related pathways in just one astrocyte subtype among people with major depression, bipolar disorder, schizoaffective disorder, or schizophrenia who had experienced severe childhood adversity (Kaul et al., 2024). Such spatially resolved, genome scale views show that stress does not uniformly alter all cell types, but that there are discrete cellular populations that are particularly vulnerable to the effects of stress, providing new insight into highly specific therapeutic targets.

Given the cell-type specificity of neuropeptide expression in the brain, it is critical to be able to analyse their spatial distribution. Importantly, proof-of-concept work shows this to be entirely possible. Specifically, in rats, vasopressin and oxytocin transcripts display striking sex specific topographies within the fresh frozen supraoptic nucleus (Nguyen et al., 2023), while aging female mice reveal discrete hypothalamic neuron clusters enriched for neuropeptide mRNAs (Hajdarovic et al., 2022). Human data echo this spatial precision: in the fresh frozen locus coeruleus, neuropeptide genes occupy sharply defined micro domains (Weber et al., 2024), and in Alzheimer's disease, somatostatin expression in the inferior temporal cortex falls off specifically in the immediate halo around amyloid-β plaques (Kwon et al., 2023). Collectively, these findings demonstrate that neuropeptide transcripts are not uniformly distributed but are instead enriched in highly localised circuits that are vulnerable to stress and neurodegeneration. This makes them feasible novel targets for drug development.

Emerging spatial maps of the human brain at subcellular resolution further support that neuropeptide circuits are organised with astonishing cellular precision. In the dorsal striatum, for example, integrating wide-field and single-cell datasets uncovered eight major interneuron classes and fourteen finer subclasses, each with distinctive neuropeptide transcriptional signatures (Garma et al., 2024). Likewise, sex-biased gene programs in the ventromedial and arcuate nuclei of the hypothalamus were segregated into sharply defined cellular niches (Mulvey et al., 2024). These findings show that neuropeptide expression is not only region-specific but also confined to molecularly unique cell-types and even subcellular territories. These patterns are likely reshaped by stress and in psychiatric illness, and therefore, present as promising entry points for targeted therapies.

While transcriptomic maps have begun to chart where neuropeptide genes are switched on or off, neuroactive steroids pose a different problem: they arise from cholesterol, not direct gene transcription (Fig. 2). To study the upstream regulatory networks that link the two molecular classes, we need to be able to study them across the same spatial and temporal planes. The steroidogenic enzymes that generate neurosteroids are themselves tightly regulated at the mRNA level (Table 1), offering an indirect but highly informative window into neurosteroid signalling. Spatial profiling of these enzymes has already proven its value: visualising *Cyp17a1* transcripts in mouse ovary, for instance, cleanly separated androgen producing thecal cells from adjacent granulosa cells (Luo et al., 2024). In the brain, enzyme expression varies sharply across regions and cell-types, hinting at equally fine-grained control over local steroid production (Melcangi et al., 1998; King et al., 2002; Agís-Balboa et al., 2006; Do Rego et al., 2009; Reddy, 2010; Taves et al., 2011; Locci and Pinna, 2017; Lin et al., 2022). High-resolution spatial analysis of these transcripts could, therefore, expose previously hidden patterns of neurosteroid synthesis and dysregulation, refining our understanding of how cholesterol derived modulators shape (and sometimes derail) neuronal circuits in health and psychiatric disease.

### 6.2 Integrating spatial proteomics with transcriptomics offers the next level of regulatory resolution

Transcript maps, no matter how sharp, capture only one layer of regulation. Neuroactive steroids are products, not transcripts, and most active neuropeptides emerge only after proteolytic processing and other post translational modifications. Since disease often perturbs several regulatory tiers at once, a full picture of homeostatic or pathological neurobiology demands that we look beyond RNA, instead to the proteins and metabolites themselves. Integrating spatial proteomics with the transcriptomic frameworks outlined above offers that next layer of resolution. Commercially available workflows that pair RNA and protein mapping make this feasible, though current immuno-based chemistries still favour predefined target panels. Expanding these panels or coupling them with unbiased mass-spectrometry-derived spatial proteomics will be essential for capturing the complete repertoire of steroidogenic enzymes, processed neuropeptides, and their receptors within the same cellular neighbourhoods, thereby illuminating multi-layered dysregulation in stress-linked brain disorders.

A recent illustration of multi-layer spatial profiling comes from Schafer et al. (2023), who interrogated microglial ageing in the female mouse hippocampus using an integrated RNA-plus-protein pipeline. By layering a 950-plex neuroscience RNA panel and a 50-gene senescence module onto antibody markers for histones, rRNA, DAPI (nuclear marker) and glial fibrillary acidic protein (GFAP, astrocytes), they traced microglial phenotypes at single-cell resolution in formalin-fixed tissue. The hippocampal white matter emerged as a hotspot where microglia progressively shift identity with age. Importantly, the authors had predicted that senescent, disease-associated microglia would co-express *Cdkn2a/p16*^*ink4a*^ and *Lgals3*, but the spatially resolved data showed these markers occupy adjacent yet distinct microglial populations that only interact, rather than converge within the same cells (Schafer et al., 2023). This detailed dissection of transcript and protein landscapes exemplifies how spatial multiomics can overturn assumptions drawn from bulk or single-modality datasets and pinpoint cell-state transitions that may underlie vulnerability to neuropsychiatric phenotypes.

Another investigation in a male mouse model of Alzheimer's disease underscores the same principle. Mapping hippocampal and adjacent cortical tissue, Mallach et al. (2024) combined a high-plex neuroscience RNA panel with antibodies against histones, DAPI, GFAP, and the amyloid-β marker MOAB-2. By first delineating the plaque core in protein space and then querying the transcriptome within and around that niche with spatial transcriptomics, they showed that microglia are strikingly enriched and transcriptionally re-programmed at amyloid deposits (Mallach et al., 2024). This ability to anchor gene expression to precisely defined protein abnormalities demonstrate a key strength of multi-layer spatial omics. Questions can be asked not just about “where is a transcript?” but “what does the transcriptome look like exactly at the point of a pathogenic protein?”, or “how is gene expression related to protein expression with anatomical context?” Both the Schafer and Mallach studies therefore demonstrate how paired transcript-proteome maps deliver sub-cellular, three-dimensional insight into cell-state changes that would be blurred or entirely lost if RNA and protein were measured in separate tissue sections. For complex and highly heterogeneous organs such as the human brain, retaining this integrated spatial context is crucial for disentangling the layered molecular networks that go awry in stress-related and neurodegenerative disorders.

While incredibly informative, platforms capable of producing high-resolution transcript-proteome maps are still currently limited by price. Additionally, they allow only the transcriptomic analysis of neuropeptides and steroidogenic enzymes as neuroactive steroids are not direct gene products, but rather metabolites of cholesterol (Reddy, 2010). While transcriptomics can answer how the expression of genes which encode neuropeptide precursors are changing, it falls short in addressing how post-translational modifications of these precursors (capable of producing multiple pleiotropic peptide products) affect neuropeptide localisation in relation to the diverse cytoarchitecture of the human brain. On the other hand, proteomics with immuno-based detection methods such as in CosMx, are capable of neuropeptide detection using antibodies, but there are also limitations to its application because of the similarity in neuropeptide chemical structure (Delaney et al., 2018). For example, epitopes, the regions of an antigen (target molecule) that bind to an antibody are usually ∼5–21 amino acids long (Delves et al., 2017). Neuropeptides typically range ∼3–100 amino acids long, and for some, only differ in a small proportion of their amino acid sequence. One of the most well-known examples of this is vasopressin and oxytocin, each of which are 9 amino acids long with 7 residues identical between the two (Mains and Eipper, 1999). Other limitations applicable to both neuroactive steroids and neuropeptides alike is rapid degradation (Balthazart and Ball, 2006; Schrader et al., 2014), low *in vivo* concentrations (Gomez-Sanchez et al., 2005; Romanova and Sweedler, 2015), and high rates of delocalisation during either lengthy and/or repetitive wash steps (Hulme et al., 2020).

### 6.3 Integrating the unbiased detection of proteins, peptides, lipids and metabolites using mass spectrometry imaging for characterising the multi-layered dysregulation common in stress-related brain disorders

A recent study by Vicari et al. (2023) demonstrates a highly informative multimodal approach to tissue analysis that combines histology, mass spectrometry and spatial transcriptomics to give a holistic overview of mRNA transcripts, proteins, lipids and low molecular weight metabolites across a single tissue sample. Theoretically, this technique could be used to study neurosteroids and neuropeptides in parallel, as it is not transcript dependent and can accurately discriminate between highly similar molecules. This is critical if we are to comprehensively probe both the temporal and spatial planes in which neuroactive steroids, regulated by altered neuropeptide expression (Ubuka and Tsutsui, 2022), cell-type specifically interact with DNA to modulate cellular gene expression in response to physiological stress (Lopez et al., 2021; Cruceanu et al., 2022; Rahman and McGowan, 2022; Kos et al., 2023; Dony et al., 2025).

Vicari et al. integrated spatial transcriptomics (Visium platform) with matrix assisted laser desorption/ionisation mass spectrometry imaging (MALDI-MSI) of neurotransmitters in fresh frozen tissue samples from both human and mouse brain tissue in the context of dopamine-related Parkinson's disease (Vicari et al., 2023). The resulting spatially resolved data can be analysed with advanced methods such as unsupervised spatial clustering, cell-type deconvolution, and multi-sample integration, which is often supplemented by corresponding single nucleus RNA sequencing data (Long et al., 2023). A key component of the MALDI-MSI workflow is application of a matrix layer, which during the laser-based ionisation process, promotes ionisation of target species while protecting the underlying tissue for future histological analysis (Aichler and Walch, 2015). Together, spatial transcriptomics, MALDI-MSI and histological techniques such as hematoxylin and eosin (H&E) staining, or immunofluorescence, can be used to determine architectural features of the tissue section including relevant subregions, cortical layers or cell-types (Vicari et al., 2023).

MALDI-MSI could therefore represent a valuable technique in the study of stress physiology, and its contribution to psychiatric illness aetiology. While MSI analysis has been successfully applied to neuropeptides in pituitary tissues (Altelaar et al., 2007), as well as in non-human primate and rodent brains (Hummon et al., 2003; Chen et al., 2016; Hulme et al., 2020; Li and Larsen, 2023; Phetsanthad et al., 2023; Wu et al., 2023), its application in the human brain, particularly for the study of stress, remains largely unexplored. For steroids, especially glucocorticoids, MALDI-MSI has been applied to lipid-rich peripheral tissues such as the adrenal glands (Takeo et al., 2019) and serum (Nakamura et al., 2020). However, to the best of our knowledge, this technique has not yet been used to investigate these compounds in human brain tissue.

Although promising, given the low abundance of neuroactive steroids in the brain at <20 ng/g (Baulieu, 1998), direct detection at small spatial scales remains a significant technical challenge. While analysis of neurosteroids in the brain using liquid chromatography-mass spectrometry, gas chromatography-electron ionisation-mass spectrometry or negative chemical ionisation-gas chromatography-mass spectrometry has been well established for decades (see Wang et al., 2007), these techniques lack any spatial resolution. Thus MALDI-MSI may be leveraged as a novel analysis technique for the spatial analysis of neuroactive steroids directly in brain tissue, albeit its application is still in early development.

## 7 Summary and future directions in the context of physiological stress and stress-related psychiatric illness

To date, the field of neurobiology has focused predominantly on targeted analysis of protein or gene expression to answer specific biological questions. However, this approach masks changes to the regulatory networks which contribute to the expression of specific molecular targets. Despite comprising the necessary cellular machinery, stress does not impact all cells in the same way. In fact, there is increasingly convincing evidence that stress elicits a cell-(sub)type specific response (Lopez et al., 2021; Cruceanu et al., 2022; Kaul et al., 2022; Matosin et al., 2023). The complexity of this cell-specific system, especially in the context of psychiatric illness, cannot be comprehensively understood by looking at any one isolated aspect, no matter the resolution. Psychiatric illness disrupts several regulatory levels at once (i.e. from the level of the gene to protein products), therefore, investigating only one layer of this regulation will never give a full picture of the underlying homeostatic or pathological neurobiology, especially in the context of subjective environmental stimuli like stress. Multimodal application of spatially resolved analysis techniques, including transcriptomics, proteomics and imaging modalities, alternatively allows for a high-resolution overview of how these stress-sensitive regulatory networks function at a sex- and cell-type specific level.

It is worth noting that successful application of multimodal analysis techniques can also help mitigate uncertainty surrounding translation of mRNA to proteins, and the potential degrading effects the process of death and tissue storage has on mRNA. Further, they may help to circumvent limitations specific to any one technology, for example the lack of transcripts specific to neuroactive steroids, or the diverse post-translational modifications involved in neuropeptide activation. They can also start to close methodological gaps which may have previously obscured identification of specific cell populations that are particularly involved in the modulation of neurotransmission and/or sensitivity to stress. Although further development is required before MALDI-MSI can be applied to the analysis of neuroactive steroids directly in archived human brain tissue, its successful application in other lipid-rich organs, including the adrenal glands, is promising (Takeo et al., 2019).

Furthermore, in cases where resources are limited, the analysis of freely available open-source datasets, such as the Allen Brain Map (https://portal.brain-map.org/) or The Human Protein Atlas–Brain (https://www.proteinatlas.org/humanproteome/brain), provide excellent opportunities for exploratory analysis and hypothesis generation. Several stress-related datasets from published studies are also freely available, for example from Lopez et al. (2021) who published their single-cell RNA sequencing data from the PVN, pituitary and adrenal tissues obtained from both unstressed and chronically stressed mice. Leveraging existing data is a necessary and powerful approach for bridging gaps and facilitating new insights into brain function and dysfunction, especially in the context of complex neurobiological processes such as cell-type specific stress regulation and psychiatric phenotypes. Taken together, it is the comprehensive understanding of stress-regulatory systems in the brain which generates clinical relevance for the continued investigation of stress as a risk factor for psychiatric illness. Ultimately, it informs future drug development, and perhaps, even cell-type specific targets for better precision medicine approaches and therefore outcomes for patients, moving forward.

## 8 Conclusions

The intricate roles of neuroactive steroids and neuropeptides in stress regulation and psychiatric illness require a more nuanced understanding that extends beyond traditional bulk tissue analyses. While advances in spatial transcriptomics, proteomics, and MSI have enabled high-resolution mapping of gene and protein expression across specific brain regions and cell-types, gaps remain in our ability to fully capture the complexity of neuroactive steroid localisation and neuropeptide post-translational modifications across complex regulatory networks in the context of disease. In particular, the role of sex and temporal differences, need to be further considered. The application of multiomic, spatially resolved techniques offers an exciting opportunity to bridge these gaps, providing a more comprehensive multi-layered regulatory overview of the molecular interactions that underpin stress and psychiatric phenotypes. However, challenges persist in translating findings across species and ensuring the accurate localisation of neuropeptides and their metabolites, especially in the human brain. To overcome these limitations, continued integration of multiple high-resolution platforms, alongside validation using classic histological methods, will be essential. Additionally, leveraging existing open-source datasets from previously published studies and resources including the Allen Brain Map and The Human Protein Atlas could further aid exploratory research. Ultimately, expanding our mechanistic understanding of these molecular regulators will not only improve our knowledge of stress-related neurobiological disorders but will also lead to the development of more precise and effective therapeutic strategies for improved patient outcomes.

## CRediT authorship contribution statement

**Katrina Z. Edmond:** Writing – review & editing, Writing – original draft, Visualization, Conceptualization. **Natalie Matosin:** Writing – review & editing, Supervision, Funding acquisition, Data curation.

## Funding

Dr Matosin was supported by the Al & Val Rosenstrauss Fellowship provided by the Rebecca L. Cooper 10.13039/501100009187Medical Research Foundation (F2021971), the Australian National Health and Medical Research Council (10.13039/501100000925NHMRC) (APP2031881) and the 10.13039/100031212Network of European Funding for Neuroscience Research (ERA-NET 10.13039/100031366NEURON) (JTC2023).

## Declaration of competing interest

The authors declare that they have no known competing financial interests or personal relationships that could have appeared to influence the work reported in this paper.
